# RETRACTED: Mahmoud et al. Curcumin-Injected *Musca domestica* Larval Hemolymph: Cecropin Upregulation and Potential Anticancer Effect. *Molecules* 2022, *27*, 1570

**DOI:** 10.3390/molecules30183707

**Published:** 2025-09-12

**Authors:** Shaymaa Mahmoud, Sobhy Hassab El-Nabi, Asmaa Hawash, Hesham R. El-Seedi, Shaden A. M. Khalifa, Sami Ullah, Abdullah G. Al-Sehemi, Islam M. El-Garawani

**Affiliations:** 1 Department of Zoology, Faculty of Science, Menoufia University, Menoufia 32511, Egyptdrsobhyhassabelnabi@science.menofia.edu.eg (S.H.E.-N.); asmaa.khalil@su.edu.eg (A.H.); 2 Department of Bioscience, Faculty of Dentistry, Sinai University, Ismailia 41632, Egypt; 3 Department of Chemistry, Faculty of Science, Menoufia University, Menoufia 32511, Egypt; 4 International Joint Research Laboratory of Intelligent Agriculture and Agri-Products Processing, Jiangsu University, Zhenjiang 212013, China; 5 School of Food and Biological Engineering, Jiangsu University, Zhenjiang 212013, China; 6 Department of Molecular Biosciences, The Wenner-Gren Institute, Stockholm University, S-106 91 Stockholm, Sweden; shaden.khalifa.2014@gmail.com; 7 Research Center for Advanced Materials Science (RCAMS), King Khalid University, P.O. Box 9004, Abha 61413, Saudi Arabia; samiali@kku.edu.sa (S.U.); agsehemi@kku.edu.sa (A.G.A.-S.); 8 Department of Chemistry, College of Science, King Khalid University, P.O. Box 9004, Abha 61413, Saudi Arabia

The journal retracts the article titled “Curcumin-Injected *Musca domestica* Larval Hemolymph: Cecropin Upregulation and Potential Anticancer Effect” [1], cited above. 

Following publication, concerns have been brought to the attention of the Editorial Office related to image irregularities contained within this article [1].

Adhering to our standard procedure, an investigation was conducted by the Editorial Office that confirmed unusual image anomalies and duplication between Figure 3 and Figure 4 [1], presenting different experimental conditions. Following a review of the material provided by the authors, the Editorial Board has lost confidence in the integrity of the findings and has decided to retract this publication [1], as per MDPI’s retraction policy (https://www.mdpi.com/ethics#_bookmark30).

This retraction was approved by the Editor-in-Chief of the *Molecules* journal. 

The authors did not agree to this retraction.
